# Dielectric dual-dimer metasurface for enhanced mid-infrared chiral sensing under both excitation modes

**DOI:** 10.1515/nanoph-2023-0128

**Published:** 2023-05-03

**Authors:** Jingyan Li, Longfang Ye

**Affiliations:** School of Electronic Science and Engineering, Xiamen University, Xiamen 361005, China; Shenzhen Research Institute of Xiamen University, Shenzhen 518057, China

**Keywords:** chiral sensing, chirality (*C*), dielectric metasurface, rotational optical dispersion (ORD), vibrational circular dichroism (VCD)

## Abstract

Chirality (*C*) is a fundamental symmetry property of objects. Detecting and distinguishing molecular chirality in the infrared spectrum is important in life sciences, biology, and chemistry. In this paper, we demonstrate an achiral metasurface based on a gaped dual-germanium-dimer array for enhanced mid-infrared chiral sensing under both circularly polarized light (CPL) and linearly polarized light (LPL) excitations. With the metasurface, strong electric and magnetic dipole resonances with large field enhancement can be generated, resulting in an accessible superchiral hotspot in the dimer gaps under both excitation modes. The maximum electric and magnetic field enhancements exceed 220 and 100 for the bare metasurface, and exceed 70 and 60 for the metasurface coated with a 50 nm chiral biolayer under both excitations, respectively. Importantly, a high volume-averaged *C* enhancement *C*_*E_ave*_ of 241 (444) and *C*_*E_ave_bio*_ of 161 (102) under CPL (LPL) excitation can be achieved for the bare metasurface and it coated with the chiral biolayer, respectively. These results may open up new possibilities for ultrasensitive vibrational circular dichroism (VCD) and rotational optical dispersion (ORD) spectroscopy in the mid-infrared range.

## 1 Introduction

Chirality (*C*) is a fundamental property of a substance, which cannot overlap with its mirror image by translational and rotational operations [1–3]. The mirror images of chiral structures are enantiomers, which are usually found in various macro/micro structures that form the basic building blocks of life, such as nucleic acids, enzymes, amino acids, proteins, alkaloids, sugars, and carbohydrates [1, 4], [5], [6]. Although the molecular weight, density, and transition frequency of enantiomers are identical, their chemical functionality is often different [7]. The misuse of left or right enantiomers of chiral biomolecules may have some inactivating or even toxic effects on cells, leading to many diseases such as short-limb malformations, Parkinson’s disease, Alzheimer’s disease, and Huntington’s disease [8–10]. Therefore, chiral enantiomeric separation plays a key role in biology, pharmacology, toxicology, and pharmacokinetics [11, 12].

The electromagnetic interaction of chiral materials produces two effects: circular dichroism (CD) and rotational optical dispersion (ORD) [13, 14]. CD spectroscopy shows the absorption differences between left-handed circularly polarized light (LCP) and right-handed circularly polarized light (RCP). ORD can cause a rotation of the polarisation direction of the linearly polarized light (LPL). These chiral responses can be used to differentiate enantiomers. Over the past decades, CD spectroscopy has developed into an effective tool for interpreting chiral molecular information [15]. As a counterpart of CD spectroscopy, vibrational circular dichroism (VCD) spectroscopy is an equally useful technique for determining chiral molecules in the mid-infrared range [16, 17]. However, VCD and ORD signals in the mid-infrared are extremely weak that is three-order smaller than those in the visible and ultraviolet range [16–18]. The high-sensitivity chiral sensing remains a fundamental challenge.

Recent developments in metasurfaces have provided a new platform for enhancing the sensitivity of photonics biosensing [16, 19, 20]. It has been found that metasurfaces with strong superchiral field hotspots can greatly increase the weak CD signals of chiral molecules [19]. For example, various plasmonic metamaterials with large localized chirality enhancement (*C*_*E_max*_, up to two orders) have been designed, showing certain potential in some chiral sensing applications [11, 21], [22], [23], [24], [25], [26], [27]. However, such plasmonic nanostructures usually show non-uniform superhelical fields with opposite handedness, which constrain the volume-averaged *C* enhancement (*C*_*E_ave*_). Some metasurfaces with chiral nanostructures will induce intrinsic background chiral optical signals, which reduces the detection sensitivity of the chiral molecules [28, 29]. Recently, the emerging achiral dielectric metasurfaces demonstrate great potential in high-sensitivity chiral sensing applications [20, 30]. Compared to plasmonic metasurface, dielectric metasurface usually has smaller loss and lower thermal conductivity [31, 32]. Taking the advantage of supporting both electric and magnetic resonances, various dielectric metasurfaces with high refractive index dielectric nanoresonators [33, 34], such as silicon cylinders, holey silicon disks, biperiodic diamond disks, and titanium dioxide nanocube dimers, can generate strong superchiral field with *C*_*E_ave*_ about one or two orders of magnitude under CPL excitation in the visible and ultraviolet range [2, 18, 35]. Very recently, it has been demonstrated that strong localized superchiral hotspots can be generated in the gap of silicon nanocylinder dimer metasurface under linearly polarized visible light illumination [19, 36]. Besides, hybrid dielectric and metallic metasurfaces under CPL excitation have also been optimized for chiral sensing with enhanced sensitivity [2, 37, 38]. However, given the recent progress, most of the proposed metasurfaces are operated in the near-infrared, visible, and ultraviolet range [2, 9, 18, 35, 36, 39, 40]. They still suffer from the drawbacks of such as relatively low *C*_*E_ave*_, inaccessible superchiral hotspots, single excitation mode (CPL or LPL), etc. [2, 9, 18, 36, 39]. To further facilitate VCD and ORD spectroscopy for the extensive chiral molecules, investigation of efficient achiral dielectric metasurfaces with accessible superchiral hotspots, high *C*_*E_ave*_, and dual excitation modes in the mid-infrared range has become one of the key topics in photonics chiral sensing technology.

In this work, we demonstrate an efficient achiral dielectric metasurface with two excitation modes based on a gaped dual-germanium (Ge)-dimer array for enhanced chiral sensing in the mid-infrared range. The metasurface can provide significantly enhanced and spatially overlapped electric and magnetic fields with proper phase shifts, generating accessible strong superchiral hotspots in the dual-dimer gap with high *C*_*E_av*_ under both CPL and LPL excitations. To the best of our knowledge, this is the first metasurface design that can simultaneously provide high *C*_*E_ave*_ of 241 and 444 without coating, and *C*_*E_ave_bio*_ of 161 and 102 with the presence of a 50 nm chiral biolayer under both CPL and LPL mid-infrared excitations, respectively. Because of the simple achiral structure design, the metasurface is very convenient for large-scale fabrication and will not introduce background chiral-optical noise, which is very promising for future high-sensitivity chiral sensing applications.

## 2 Design and methods

**Figure 1:**
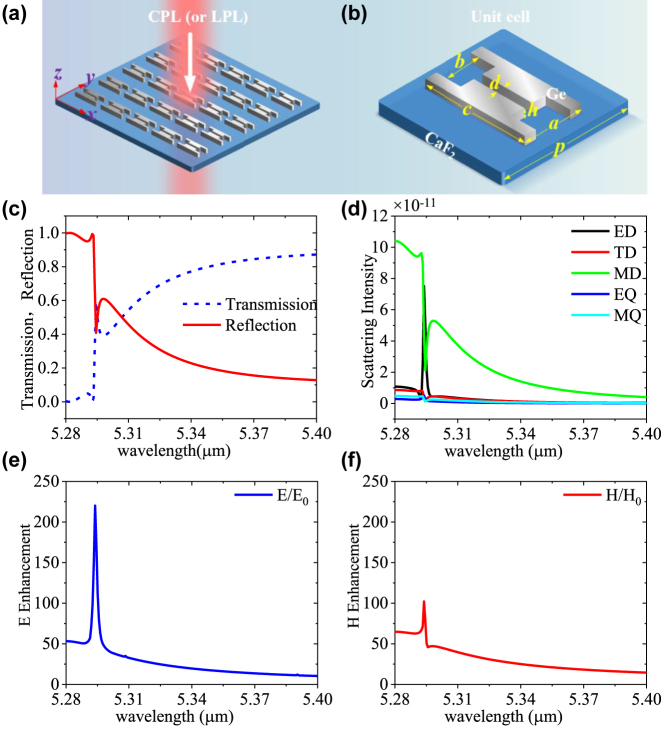
Schematic diagrams of the proposed dielectric metasurface and characteristics of the proposed metasurface under normal CPL incidence. (a) Demonstration of the dual-Ge-dimmer metasurface under normal CPL (or LPL) incidence. (b) Gaped dual-Ge-dimmer unit cell, where *a* = 0.792 μm, *b* = 0.592 μm, *c* = 3.1 μm, *d* = 0.232 μm, *h* = 1.0625 μm, *p* = 4.581 μm. (c) The transmission and reflection spectra. (d) Scattering spectra of ED, TD, MD, EQ, and MQ, respectively. (e) Electric field enhancement. (f) Magnetic field enhancement.

The proposed achiral dielectric metasurface consists of a gaped dual-Ge-dimmer array, as schematically displayed in Figure 1(a). The array is arranged in a square lattice on a CaF_2_ substrate, and the refractive indices of Ge and CaF_2_ are assumed as 4.01 and 1.39 in the target mid-infrared range [41, 42], respectively. Figure 1(b) shows the gaped dual-Ge-dimmer unit cell of the metasurface. The period *p* = 4.581 μm and other parameters are set as *a* = 0.792 μm, *b* = 0.592 μm, *c* = 3.1 μm, *d* = 0.232 μm, and *h* = 1.0625 μm. In this study, we use COMSOL Multiphysics to study the properties of the dielectric metasurface. For future experimental realization, the proposed metasurface can be fabricated by using the state-of-the-art nanofabrication technology [43] and further characterized by using the microscope-coupled Fourier transform infrared (FTIR) spectrometer [11].

In the simulation, we simulate the unit cell of the metasurface structure, where periodic boundaries are set in the *x* and *y* directions. There are two ports in the *z*-direction for the RCP or LPL normal incidence. The optical chirality *C* is calculated by [35]

(1) $$C=-\frac{\omega }{2c^{2}}Im\left(E^{*}\cdot H\right)=-\frac{\omega }{2c^{2}}\left|E\right|\left|H\right|\cos\left(\varphi_{iE,H}\right),$$

where *ω* and *c* denote the angular frequency and speed of light, **E** and **H** are the complex electric and magnetic field vectors, respectively. The chirality of a CPL $C_{\text{CPL}}=\pm \frac{\omega }{2c^{2}}E_{0}^{2}$ (+for RCP and −for LCP), where *E*_0_ and *H*_*0*_ represent the amplitude of the incident electric and magnetic fields. Considering there is no chirality for LPL, here, we define the localized *C* enhancement (*C*_*E*_) with respect to CPL for both CPL and LPL excitations as

(2) $$C_{E}=\frac{C}{C_{\text{RCP}}}=-\frac{\left|E\right|\left|H\right|\cos\left(\varphi_{iE,H}\right)}{E_{0}^{2}}$$

According to eq. (2), the condition to obtain a high *C*_*E*_ in a metasurface design includes strong electric and magnetic fields with spectrally and spatially overlapping, as well as π/2 phase.

## 3 Results and discussion

### 3.1 Characteristics of dielectric dual-dimer metasurface under CPL excitation

We first analyze the transmittance (*T*), reflectance (*R*) spectra, and field enhancement properties of the proposed dual-Ge-dimer metasurface under RCP excitation. Figure 1(c) displays the simulated transmittance (*T*) and reflectance (*R*) spectra of the metasurface in 5.28–5.40 μm. The first intersection of *T* and *R* is located at 5.294 μm. To study the resonance mechanism, we show the decomposed components of the electric dipole (ED), toroidal dipole (TD), magnetic dipole (MD), electric quadrupole (EQ), and magnetic quadrupole (MQ) resonances of the metasurface in Figure 1(d) by using the multipole decomposition methods [39, 44]. Remarkably, both ED and MD have larger values at the 5.294 μm, which satisfies Kerker’s condition [44, 45]. The simultaneous resonance of electric and magnetic dipoles strongly increases electromagnetic field strength. As shown in Figure 1(e) and (f), the maximum electric field (*E*/*E*_0_) and magnetic field (*H*/*H*_0_) enhancements at this resonance wavelength exceed 220 and 100, respectively, providing the possibility for large near-field chirality of the metasurface.

**Figure 2:**
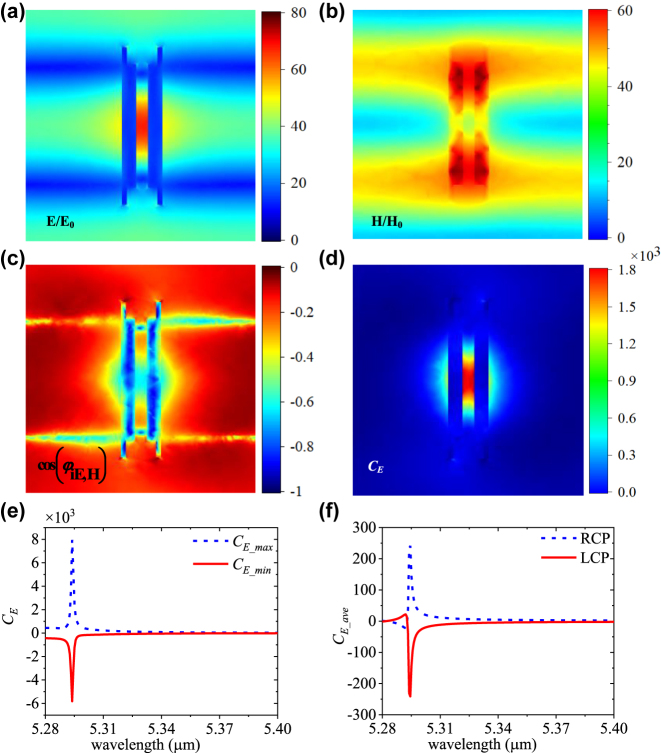
Field distributions and *C* enhancement of the dielectric metasurface under normal CPL incidence. The distributions of (a) *E*/*E*_0_, (b) *H*/*H*_0_, (c) cos(*φ*_*iE*,*H*_), and (d) *C*_*E*_ cut on *z* = *h*/2 = 0.531 μm plane of the dielectric metasurface at the wavelength of 6.594 μm. (e) The maximum and minimum *C*_*E*_ in the whole structure. (f) The volume-averaged *C*_*E_ave*_ over a 50 nm air layer upon the whole metasurface.

To better understand the electromagnetic characteristics at this point, we plot the enhancement of electric fields (*E*/*E*_0_), magnetic fields (*H*/*H*_0_), the cosine of the phase angle between **E** multiplied by the complex number *i* and **H**, cos(*φ*_*iE*,*H*_), and the localized chirality enhancement *C*_*E*_ at 6.594 μm in the *x–y* plane at *z* = *h*/2 = 0.531 μm of the dielectric metasurface in Figure 2. As shown in Figure 2(a) and (b), the enhanced electric fields are mainly distributed in the gap between two Ge dimmers, and the enhanced magnetic fields mainly are distributed inside of the two Ge dimmers, showing tens of amplitude enhancement. The enhanced *E* and *H* fields have certain special overlap around the central region of the unit cell. In Figure 2(c), we show the cos(*φ*_*iE*,*H*_) distribution on the same cut plane of the metasurface. The cos(*φ*_*iE*,*H*_) is almost negative throughout the cell region at 5.294 µm, implying the *C*_*E*_ values are mainly positive in this cutting plane (according to eq. (1)). Since the condition of the electric field, magnetic fields, as well as the phase difference, are generally satisfied (eq. (1)), high *C*_*E*_ can be obtained in the metasurface. As shown in Figure 2(d), the metasurface has superchiral fields with a single positive sign over the whole structure and a strong hotspot inside the gap between the two dimmers. Furthermore, as shown in Figure 2(e), the *C*_*E_max*_ can reach 7948 and the *C*_*E_min*_ is −5815 at 5.294 μm. To facilitate the calculation of the volume-averaged *C* enhancement, we assumed the average region is a 50 nm air layer on the metasurface. The *C*_*E_ave*_ can achieve as high as 241 and −241 under RCP and LCP illumination in Figure 2(f), respectively. The superchiral hotspot of this design is easily accessible to various chiral bio-molecules, demonstrating a promising platform for VCD spectroscopy.

**Figure 3:**
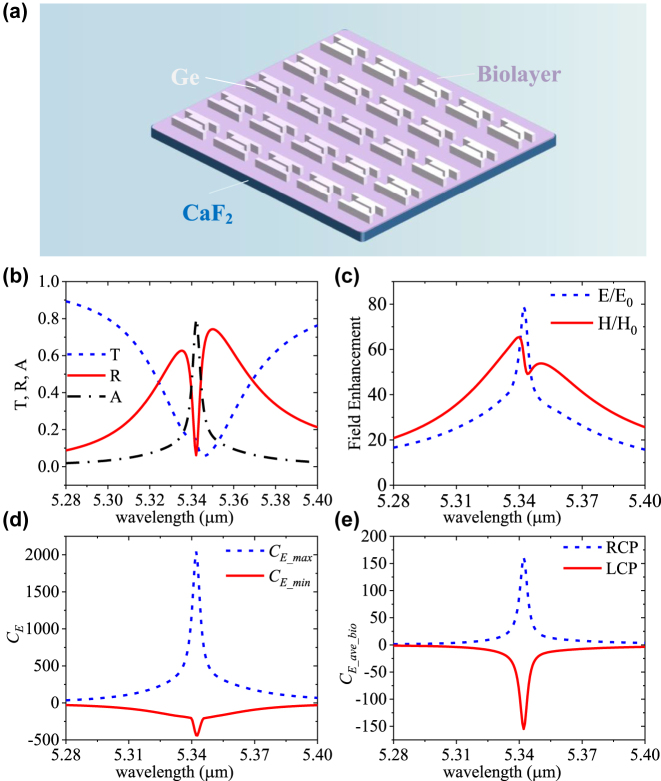
Characteristics of the proposed metasurface with 50 nm homogenous biolayer under CPL incidence. (a) Schematic diagram of the dielectric metasurface coated with a 50 nm homogenous chiral biolayer. (b) *T*, *R*, *A* spectra of the metasurface with biolayer under RCP incidence. (c) Enhancement of electric (*E*/*E*_0_) and magnetic (*H*/*H*_0_) fields. (d) The maximum and minimum *C*_*E*_ in the whole structure. (e) The *C*_*E_ave_bio*_ in biolayer volume above the metasurface under RCP (blue line) and LCP (red line) incidence.

Furthermore, to demonstrate the chiral sensing performance, a 50 nm homogenous chiral biolayer is coated above the dielectric metasurface, as shown in Figure 3(a). The refractive index of this chiral biolayer is set as *n* = 1.46 – 0.01*i* and its Pasteur parameters are set as *κ* = 0 + 0.001*i* [35]. In Figure 3(b), the *T*, *R* and absorption (*A*) of the metasurface with the chiral biolayer under RCP incidence at 5.342 μm are 0.14, 0.06, and 0.8, respectively. As shown in Figure 3(c), the enhancement of electric fields *E*/*E*_0_ and magnetic fields *H*/*H*_0_ can achieve 77 and 56 at 5.342 μm, respectively. Notably, there is a 0.048 μm spectra redshift of the metasurface with and without biolayer, as shown in Figures 3(b) and 1(c). With the chiral biolayer, the maximum values of *E*/*E*_0_ and *H*/*H*_0_ are also decreased. As shown in Figure 3(d), the *C*_*E_max*_ peak of 1969 and *C*_*E_min*_ dip of −395 at 5.342 μm are achieved. The volume-averaged *C*_*E_ave_bio*_ of the metasurface with biolayer achieves 161 under RCP illumination and −154 under LCP illumination, respectively, as shown in Figure 3(e). These values are smaller than the metasurface without the biolayer (±241). Nevertheless, the chirality enhancement |*C*_*E_ave_bio*_| is still much higher than most of the previously reported work [2, 18, 19, 32, 35], showing great potential applications in high-sensitivity chiral sensing. In addition, the impact of the geometric parameters *b* and *d* on the *C*_*E_ave_bio*_ of the metasurface is discussed in the Supporting Information (Figure S1).

### 3.2 Characteristics of dielectric dual-dimer metasurface under LPL excitation

**Figure 4:**
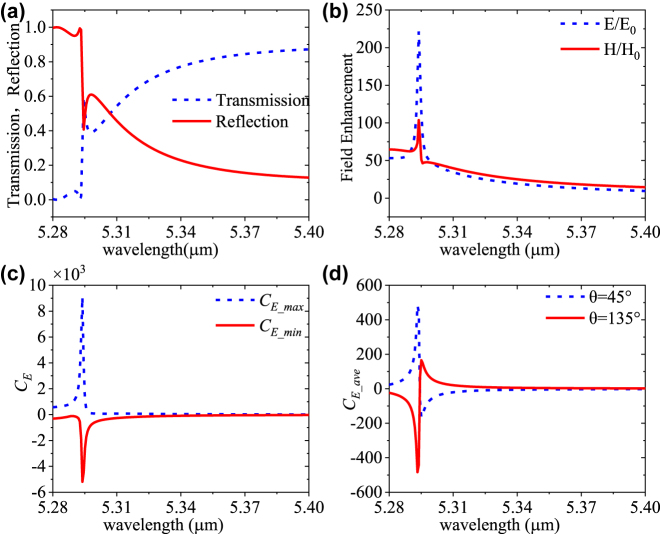
Characteristics of the proposed metasurface under normal LPL incidence. (a) *T*, *R*, and *A* spectra of the metasurface. (b) Enhancement of electric (*E*/*E*_0_) and magnetic (*H*/*H*_0_) fields. (c) The maximum and minimum *C*_*E*_ in the whole structure. (d) The *C*_*E_ave*_ in air layer volume above the metasurface under LPL.

The proposed dielectric metasurface can also be used to distinguish chiral molecules using optical rotatory dispersion (ORD). ORD can cause the rotation of the polarization direction from LPL. Here, instead of the CPL excitation, LPL with the polarization angle *θ* = 45° and 135° is subsequently used for the dual-Ge-dimer metasurface excitation. The simulated *T* and *R* spectra under 45° LPL are shown in Figure 4(a). The *T* and *R* spectra under 45°/135° LPL and LCR/RCP are identical. As shown in Figure 4(b), this design can achieve high enhancements of electric field (*E*/*E*_0_) and magnetic field (*H*/*H*_0_) of more than 221 and 103 at around 5.293 μm, respectively. Figure 4(c) shows the calculated maximum and minimum localized *C*_*E*_ in the whole structure can achieve 9090 and −5189 at 5.293 μm, respectively. Figure 4(d) shows that the volume-averaged *C*_*E_ave*_ in the 50 nm air layer above the structure reaches ±444 at 5.293 μm (and ∓165 at 5.295 μm) with the polarization angle *θ* = 45° and 135°, respectively. The *E*/*E*_0_, *H*/*H*_0_, *C*_*E_max,*_ and *C*_*E_ave*_ of the metasurface under LPL are higher than those under RCP incidence. Considering the *C*_*E_ave*_ jumps from positive to negative from 5.293 to 5.295 μm, the metasurface shows a narrowband chiral sensing characteristic under LPL excitation. Moreover, the characteristics of *E*/*E*_0_, *H*/*H*_0_, cos(*φ*_*iE*,*H*_), and *C*_*E*_ of the metasurface under LPL excitation are similar to those under RCP incidence (Figure 2), as shown in Figure S2 in the Supporting Information.

**Figure 5:**
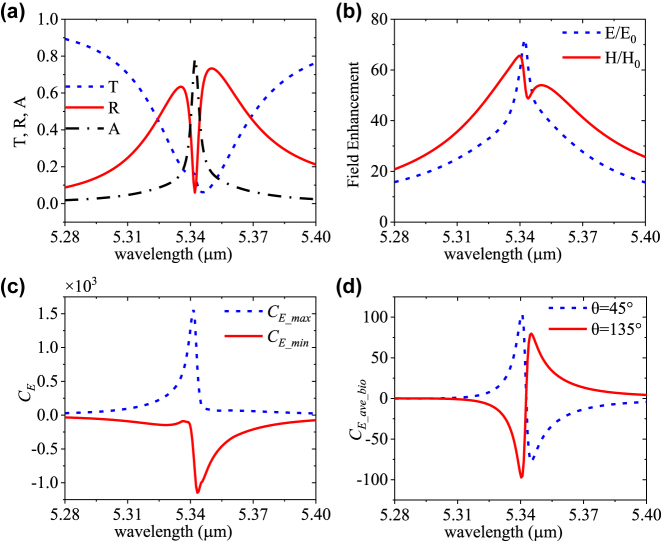
Characteristics of the proposed metasurface with 50 nm homogenous biolayer under LPL incidence. (a) *T*, *R*, and *A* spectra. (b) Field enhancement *E*/*E*_0_ and *H*/*H*_0_. (c) The maximum and minimum *C*_*E*_ in the whole structure. (d) The *C*_*E_ave_bio*_ in the biolayer volume above the metasurface under LP incidence.

Furthermore, we study the performance of the dual-Ge-dimer metasurface covered with the same 50 nm chiral biolayer under LPL excitation. Figure 5(a) shows its *T*, *R*, and *A* spectra, which are also similar to the RCP excitation case in Figure 3(b). Figure 5(b) shows that the *E*/*E*_0_ and *H*/*H*_0_ reach 65 and 64 at 5.34 μm, respectively. Figure 5(c) shows that the *C*_*E_max*_ and *C*_*E_min*_ are 1504 and −373 at this point. The *C*_*E_ave_bio*_ achieves a very large value of 102 at 5.34 μm and −77 at 5.34 μm, as shown in Figure 5(d), demonstrating great capability in chiral sensing under LPL excitation.

### 3.3 Performance comparison of dielectric metasurfaces for chiral sensing

Finally, we compare the performance of the proposed and the recently reported dielectric metasurfaces for chiral sensing in Table 1. For CPL excitation, the silicon cylinder metasurface shows *C*_*E_ave*_ of 6.5 [33]. The holey silicon disk metasurface shows *C*_*E_ave*_ of 45 and *C*_*E_ave_bio*_ of 24 in visible [35]. The biperiodic diamond disk metasurface supports a very high localized *C*_*E_max*_ of 1130 and *C*_*E_ave*_ of 100 in a cut-plane in the ultraviolet [18]. The SiO_2_ nanocube dimer [2] and the quasi-BIC TiO_2_ dimer [38] can achieve a large *C*_*E_ave*_ of 50 in the central region and 59 in the whole region, respectively. The hollow Si disk achieves *C*_*E_ave_bio*_ of 12 in the whole region [39] and the nanodisk produces *C*_*E_ave*_ of 50 in the whole region [34]. For the LPL excitation, the silicon nanocylinder dimer metasurface realizes a high *C*_*E_max*_ of 300, *C*_*E_ave*_ of 180 and *C*_*E_ave_bio*_ of 120 in the central region [19]_._ However, all of these metasurfaces operate only in a single CPL or LPL excitation mode. In this work, the dual-Ge-dimer metasurface achieves better optical chiral enhancement operating in both excitation modes, where *C*_*E_ave*_ of 241 and *C*_*E_ave_bio*_ of 161 for the CPL excitation, and *C*_*E_ave*_ of 444 and *C*_*E_ave_bio*_ of 102 for the LPL excitation, providing great potential applications in high-efficiency and high-sensitivity chiral sensing in the mid-infrared range.

**Table 1:** Comparison of the proposed and the reported dielectric metasurfaces for chiral sensing.

Years (refs.)	Structures	Materials	Wavelengths	Polarizations	*C*_*E_max*_	*C*_*E_ave*_/region	*C*_*E_ave_bio*_/region
2019 [32]	Cylinder	Si	1.8–3.9 μm	CPL	–	6.5/whole	–
2019 [35]	Holey disk	Si	600–1200 nm	CPL	–	45/whole	24/whole
2019 [18]	Biperiodic disk	Diamond	255–270 nm	CPL	1130	100/cut-plane	–
2019 [2]	Nanocube dimer	SiO_2_	350–420 nm	CPL	120	50/central	7.5/central
2020 [38]	Quasi-BIC dimer	TiO_2_	845–875 nm	CPL	247	59/whole	–
2021 [39]	Hollow disk	Si	500–1500 nm	CPL	39	–	12/whole
2022 [34]	Nanodisk	Si	1.0–1.6 nm	CPL	138	30/whole	–
2022 [19]	Nanocylinder dimer	Si	450–650 nm	LPL	300	180/central	120/central
This work	Gaped dual-dimer	Ge	5.28–5.4 μm	CPL; LPL	5280; 9090	241/whole; 444/whole	161/whole; 102/whole

## 4 Conclusions

In summary, we present an achiral dielectric metasurface consisting of a dual-Ge-dimmer array on a CaF_2_ substrate for enhanced chiral sensing under both CPL and LPL excitations. When under RCP incidence, the metasurface supports strong electric and magnetic dipole resonance. A high volume-averaged *C*_*E_ave*_ of 241 and *C*_*E_ave_bio*_ of 161 can be achieved for the bare metasurface and the metasurface coated with a 50 nm chiral biolayer. While under the LPL excitation, high field enhancement and the volume-averaged *C*_*E_ave*_ of 444, *C*_*E_ave_bio*_ of 102 can be obtained for the bare metasurface and the metasurface coated with a 50 nm chiral biolayer. Remarkably, the metasurface supports single-handedness superchiral fields with a strong hotspot in the gap between the two dimmers for both excitation modes, which can be easily accessible to various chiral bio-molecules. The simple achiral structure makes it free of structural chiral noise and easy for large-scale fabrication, which is very promising for future high-efficiency and high-sensitivity chiral sensing applications.

## Supporting information

1. Impact of the geometric parameters b and d on the chirality enhancement of dielectric dual-dimmer metasurface under both CPL and LPL excitations.
2. Simulated field distribution of dielectric dual-dimer metasurface under LPL excitation.

## Supplementary Material

Supplementary Material Details
