# Supplementary material for: Dielectric dual-dimer metasurface for enhanced mid-infrared chiral sensing under both excitation modes
Source: Nanophotonics. 2023 May 3;12(12):2189–97. doi: 10.1515/nanoph-2023-0128 (PMC11501344; doi:10.1515/nanoph-2023-0128)
Supplement: Supplementary file 1 — Supplementary Material Details [file j_nanoph-2023-0128_suppl_001.docx]

Supporting information

**Dielectric dual-dimer metasurface for enhanced mid-infrared chiral sensing under both excitation modes**

**Jingyan Li^1,2^ and Longfang Ye^1,2^**^✉^

^1^School of Electronic Science and Engineering, Xiamen University, Xiamen 361005, China

^2^Shenzhen Research Institute of Xiamen University, Shenzhen 518057, China

^✉^**Corresponding author email:** [lfye@xmu.edu.cn](mailto:lfye@xmu.edu.cn)

1. **Impact of the geometric parameters *b* and *d* on the chirality enhancement of dielectric dual-dimmer metasurface under both CPL and LPL excitations**

The chirality enhancement of the dielectric dual-dimmer metasurface is sensitive to the varying of the structure geometric parameters, especially on the lengths *b* and *d*. This is because the chirality enhancement arises from the metasurface’s resonance, which is affected by its structural parameters. In Fig.S1, we show the dependence of *C_E__ave_bio* on *b* and *d* of the metasurface coated with a 50 nm biolayer under both RCP and LPL excitations, while keeping other parameters the same as those shown in the caption of Fig. 1(b). For the RCP excitation, as the length *b* increases from 0.492 to 0.592 and then to 0.692 μm, the peak *C_E__ave* increases from 125 to 160 and then decreases to 138 with the corresponding wavelength blueshifts from 0.3430 to 0.3414 and then to 5.3410 μm, as shown in Fig. S1 (a). As the length *d* increases from 0.212 to 0.232 and then to 0.252 μm, the *C_E__ave* increases from 145 to 160 and then decreases to 128 with the corresponding wavelength redshifts from 0.3405 to 0.3414 and then to 5.343μm, as shown in Fig. S1 (b). Furthermore, for the LPL excitation, as the length *b* increases from 0.492 to 0.692 μm, the peak *C_E_ave_bio_* increases from 38 to 135, and the corresponding wavelength blueshifts from 5.342 to 5.339 μm, as shown in Fig. S1 (c). As the length *d* increases from 0.212 to 0.252 μm, the peak *C_E_ave_bio_* decreases from 131 to 35, and the corresponding wavelength redshifts from 5.338 to 5.341 μm, as shown in Fig. S1 (d).

1. **Simulated field distribution of dielectric dual-dimer metasurface under LPL excitation**

Fig. S2 shows the electric field E/E_0_, magnetic field H/H_0_, $\cos(\varphi_{iE,H})$, and *C_E_* distributions in *z* = 0.531 μm cut plane of the dielectric metasurface under 45° LPL excitation at the wavelength of 5.293 μm. The electric field E/E_0_ with a maximum of more than 60 is mainly distributed in the gap between the two Ge dimmers. The H/H_0_ with a maximum of more than 60 is mainly concentrated inside two Ge dimmers and their gap. The $\cos(\varphi_{iE,H})$ shows a single negative sign throughout the whole cut plane, implying the *C_E_* has single positive values in this region. Therefore, distributions of E/E_0_, H/H_0_, $\cos(\varphi_{iE,H})$, and *C_E_* of the metasurface under LPL excitation are similar to those under RCP incidence (Fig. 2). Since the conditions for the electric and magnetic fields as well as the phase difference are generally satisfied (equation (1)), large *C_E_* up to 3 orders in the gap between the two Ge dimmers can be achieved.

Fig.S1 (a) The dependence of *C_E_ave_bio_* on length *b* under RCP excitation*.* (b) The dependence of *C_E_ave_bio_* on the length *d* under RCP excitation*.* (c) The dependence of *C_E_ave_bio_* on length *b* under LPL excitation*.* (d) The dependence of *C_E_ave_bio_* on the length *d* under LPL excitation*.*

Fig. S2 The distributions of (a) E/E_0_, (b) H/H_0_, (c) $\cos(\varphi_{iE,H})$, and (d) *C_E_* cut on *z* = *h*/2 = 0.531 μm plane of the dual-Ge-dimer metasurface under LPL excitation at the wavelength of 5.293 μm.
